# Decellularized Cartilage Extracellular Matrix Incorporated Silk Fibroin Hybrid Scaffolds for Endochondral Ossification Mediated Bone Regeneration

**DOI:** 10.3390/ijms22084055

**Published:** 2021-04-14

**Authors:** Vivek Jeyakumar, Nedaa Amraish, Eugenia Niculescu-Morsza, Christoph Bauer, Dieter Pahr, Stefan Nehrer

**Affiliations:** 1 Center for Regenerative Medicine, Danube University Krems, 3500 Krems, Austria; eugenia.niculescu-morzsa@donau-uni.ac.at (E.N.-M.); christoph.bauer@donau-uni.ac.at (C.B.); stefan.nehrer@donau-uni.ac.at (S.N.); 2 Department of Anatomy and Biomechanics, Karl Landsteiner University for Health Sciences, 3500 Krems, Austria; nedaa.amraish@kl.ac.at (N.A.); dieter.pahr@kl.ac.at (D.P.); 3 Institute for Lightweight Design and Structural Biomechanics, Vienna University of Technology, 1060 Vienna, Austria

**Keywords:** hybrid scaffolds, decellularized cartilage ECM, silk fibroin, endochondral ossification, bone regeneration

## Abstract

Tissue engineering strategies promote bone regeneration for large bone defects by stimulating the osteogenesis route via intramembranous ossification in engineered grafts, which upon implantation are frequently constrained by insufficient integration and functional anastomosis of vasculature from the host tissue. In this study, we developed a hybrid biomaterial incorporating decellularized cartilage extracellular matrix (CD-ECM) as a template and silk fibroin (SF) as a carrier to assess the bone regeneration capacity of bone marrow-derived mesenchymal stem cells (hBMSC’s) via the endochondral ossification (ECO) route. hBMSC’s were primed two weeks for chondrogenesis, followed by six weeks for hypertrophy onto hybrid CD-ECM/SF or SF alone scaffolds and evaluated for the mineralized matrix formation in vitro. Calcium deposition biochemically determined increased significantly from 4-8 weeks in both SF and CD-ECM/SF constructs, and retention of sGAG’s were observed only in CD-ECM/SF constructs. SEM/EDX revealed calcium and phosphate crystal localization by hBMSC’s under all conditions. Compressive modulus reached a maximum of 40 KPa after eight weeks of hypertrophic induction. μCT scanning at eight weeks indicated a cloud of denser minerals in groups after hypertrophic induction in CD-ECM/SF constructs than SF constructs. Gene expression by RT-qPCR revealed that hBMSC’s expressed hypertrophic markers VEGF, COL10, RUNX2, but the absence of early hypertrophic marker ChM1 and later hypertrophic marker TSBS1 and the presence of osteogenic markers ALPL, IBSP, OSX under all conditions. Our data indicate a new method to prime hBMSC’S into the late hypertrophic stage in vitro in mechanically stable constructs for ECO-mediated bone tissue regeneration.

## 1. Introduction

Orthopedic implants, such as prostheses, bone autografts, and allografts, are available in clinical settings to treat bone defects in non-union fractures, trauma, or osteoporosis, and have limitations due to their physical performance in healing the defects. This is a major socioeconomic problem regarding insubstantiality and weak recovery and is associated with high health care costs. In the EU only, clinical failure from non-union bone defects resulting from impaired osteogenesis with existing bone graft transplantations was estimated to be 1 million recipients in 2017. The annual number of fracture casualties is expected to rise from 2.7 million in 2017 to 3.3 million in 2030, corresponding to a 23% increase [1]. Bone autografts remain the gold standard for treating patients with bone defects and have shown promising outcomes, but they are limited by their availability and donor site morbidity. The field of bone tissue engineering (BTE) is a rapidly developing area in orthopedic regenerative medicine that focuses on alternative treatment options that will solve existing clinical treatment problems over time. Standard approaches in BTE target the route of osteogenesis, utilizing osteoinductive and osteoconductive biomaterials at the site of implantation with osteoprogenitor cells [2]. However, this strategy is primarily associated with inadequate vascularization and integration with the host tissue. Natural bone healing at the site of the defect involves mostly three vital phases. First, in a reactive phase, blood vessels come in proximity to an injury and constrict to stop the bleeding process, resulting in a clot formation, allowing fibroblasts to expand and form granular tissue. Second, in the reparative phase, fibroblasts from the granular tissue differentiate into primary chondroblasts capable of producing hyaline cartilage. Third, hyaline cartilage formation connects the gap between the fracture site and results in a callus development. The hyaline cartilage at later stages gets replaced by the lamellar bone. This process of replacement is known as endochondral ossification [3].

The current challenges faced in clinical therapies for the repair of large bone defects are to develop sustainable tissue engineered (TE) constructs that can mimic the natural structure and functional aspects of bone tissue—providing biomechanical and biological signals that can stimulate the mechanism of bone remodeling as occurring in the native state under physiological settings. Several requirements have to be fulfilled when developing clinically viable TE bone constructs, such as biocompatibility, controlled degradation, high porosity, and permeability for cells to infiltrate, vascularize, and integrate into the host when implanted [4,5]. The functional BTE paradigm highlights these TE constructs’ fulfillment to perform osteoconduction, osteoinduction, and osseointegration after host implantation, along with the functions as mentioned above.

**Figure 1:**
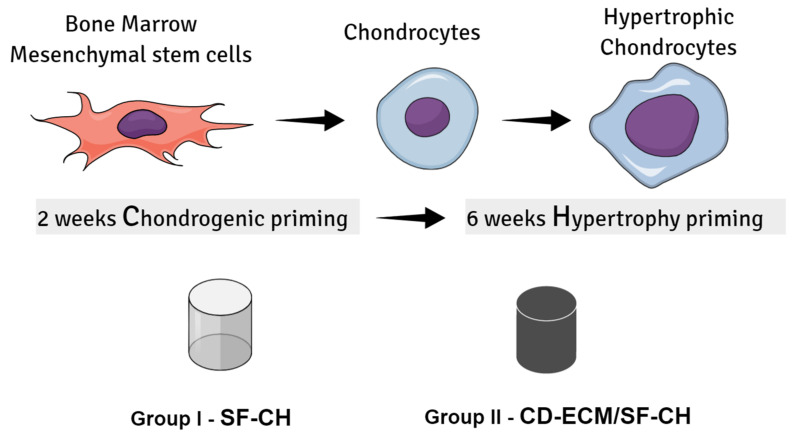
Schematic representation of the experimental design setup.

Over the last decades, substantial attention was given to understand the components of the extracellular matrix (ECM) of skeletal tissues, like bone and cartilage in the area of orthopedic regenerative medicine to mimic and design ECM-like biomaterials [6,7,8]. ECM is the core constituent of connective tissues like cartilage and bone that regulates the tissue structure’s maintenance and organization. ECM controls both mechanical strength and structural rigidity and maintains tissue homeostasis. Engineered bone based on endochondral ossification gained significant interest over the last decade [9]. The formation of long bones stimulates the idea of reverse engineering bone via endochondral ossification during embryonic development and fracture healing [10]. During long bone formation, mesenchymal stem cells (MSCs) aggregate and differentiate into chondrocytes, which then produce a cartilaginous template rich in collagen II (COLII) and glycosaminoglycans (GAGs). Eventually, the chondrocytes undergo hypertrophic differentiation and ossify into a mineralized matrix. This denotes that hypertrophic chondrocytes mediate the undeviating path for endochondral bone formation [11]. However, challenges in tissue-engineered bone constructs include functionality under the mechanical environment inside in vivo conditions. Engineered bone constructs must withstand higher mechanical loads under physiological conditions [12]. Therefore, engineered bone’s biomechanical properties are an essential factor in the development of functional grafts for clinical therapies. Our current study aimed to modulate endochondral ossification-driven mineralization by human bone marrow-derived mesenchymal stem cells (hBMSCs) in a decellularized cartilage-derived extracellular matrix (CD-ECM), incorporating silk fibroin (SF) as a hybrid scaffold in vitro. RT-qPCR determined the gene expression for hypertrophic and osteogenic markers, and to quantification of the mineralized tissue formation µCT scans was performed (Figure 1).

## 2. Results

Results were obtained by the following methodologies. Biochemical analysis determined the decellularization and mineralization post differentiation. SEM determined the morphology of the constructs. RT-qPCR determined the gene expression for hypertrophic and osteogenic markers, and, in order to quantify the mineralized tissue formation, µCT scans were performed. Biomechanical analysis determined the mechanical properties of constructs pre and post differentiation.

### 2.1. Decellularization Does Not Influence the Retention of ECM Components

**Figure 2:**
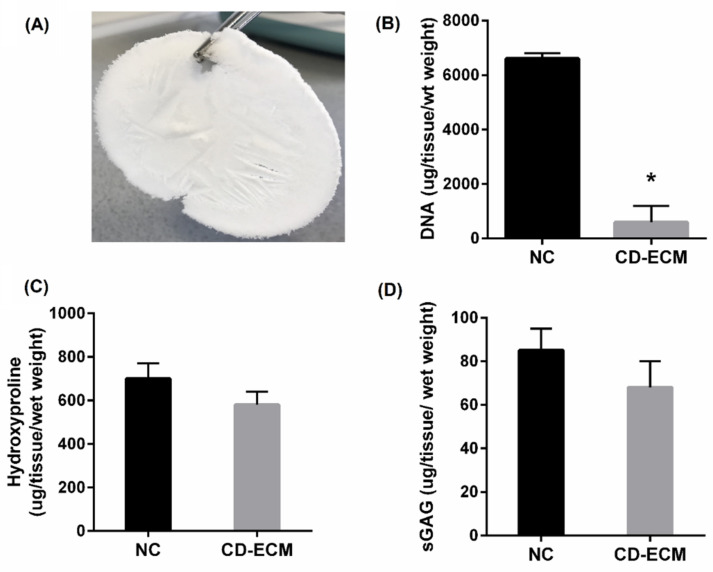
Biochemical content of cartilage post decellularization (**A**). Total dsDNA (**B**), total collagen by hydroxyproline measurement (**C**), and sulfated glycosaminoglycan) (**D**) in comparison to the native cartilage (NC). Significant difference at * *p* < 0.05; *n* = 6.

The decellularization protocol by osmotic shock, detergent washing, and nucleic acid removal with ionic exchange (Figure 2A) indicates that more than 85% of the DNA content was removed significantly (*p* < 0.05) (Figure 2B). Total collagen content determined by the hydroxyproline measurement revealed that 95% of the collagen matrix is retained compared to the native cartilage (Figure 2C) with no significant difference. Similar observation occurred in the total sulfated glycosaminoglycan (sGAG) content (Figure 2D) with no significant difference to the native cartilage tissue.

### 2.2. Porosity Can Be Controlled, and CD-ECM Is Deposited onto the Void Struts of SF Scaffolds as Hybrid Biomaterials

**Figure 3:**
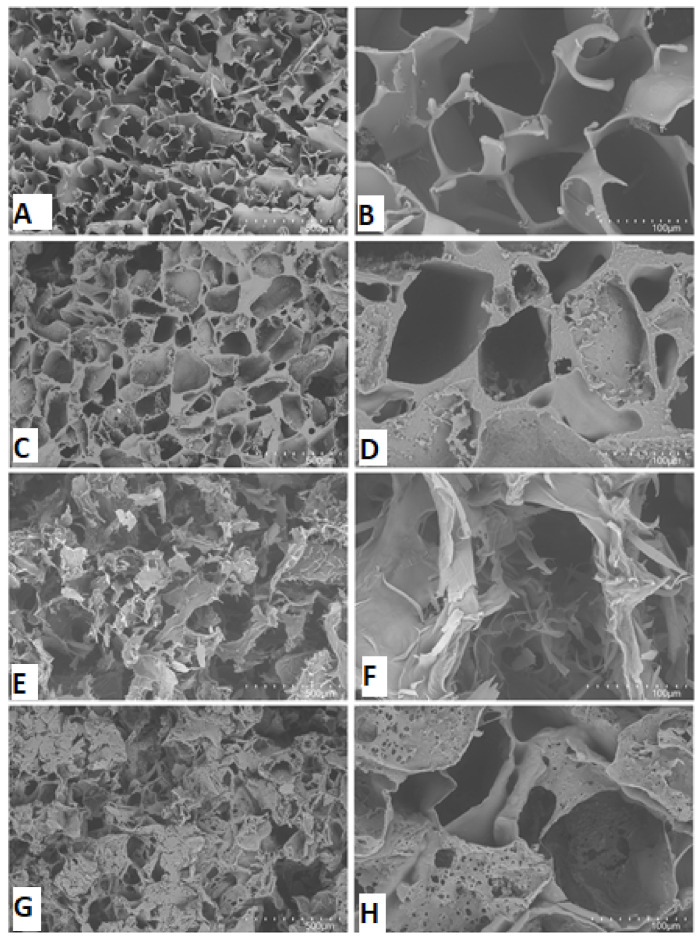
SEM-based morphological observation of SF scaffolds before (**A**,**B**) and after (**C**,**D**) methanol treatment. Incorporation of CD-ECM before (**E**,**F**) and after incorporated to CD-ECM/SF scaffolds (**G**,**H**). Scale bar represents 500 µm on left and 100 µm on right.

The solvent casting and salt-leaching resulted in maintaining the SF scaffold’s desired porosity by induction of tertiary structure formation of random coil SF (Figure 3B) to the β-sheet formation upon methanol crosslinking (Figure 3D). During the crosslinking procedure, introducing CD-ECM fibers led to CD-ECM deposition onto the void struts of CD-ECM, as observed by SEM on both longitudinal and cross-sections morphologically (Figure 3H).

**Figure 4:**
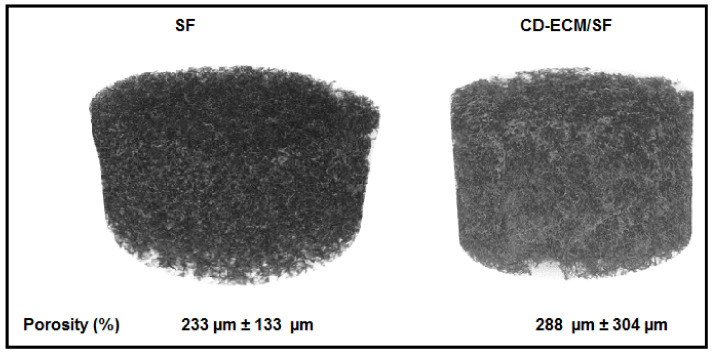
Porosity measurements empty SF (left) and CD-ECM/SF (right) scaffolds scanned by µCT with a resolution of 5 µm. Significant difference at *p* < 0.05; *n* = 15.

Porosity measurement of the entire constructs by µCT indicated a porosity of 233 µm ± 133 µm in SF scaffolds and 288 µm ± 304 µm in CD-ECM/SF hybrid scaffolds (Figure 4).

### 2.3. Methanol Crosslinking Aids Random Coil SF to Tertiary Structure and Crosslinked CD-ECM/SF Hybrid Scaffolds

**Figure 5:**
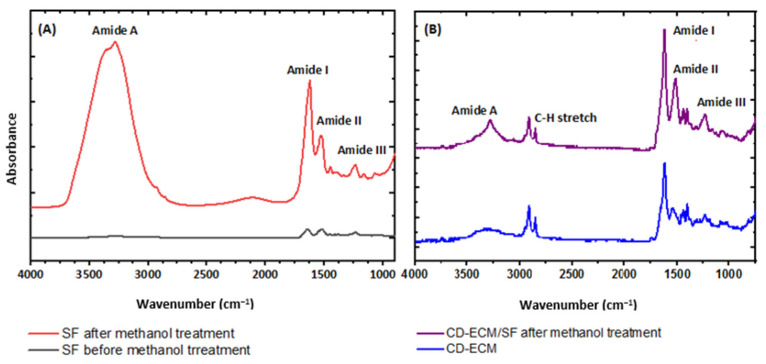
Fourier transform infrared spectroscopy (FTIR) spectrum of Silk fibroin (SF) before and after methanol treatment (**A**) and CD-ECM before and after incorporation with SF (**B**). Significant difference at *p* < 0.05; *n* = 6.

The FTIR spectra of SF scaffolds showed a N-H bend (amide II) intensity shift from 1540 cm^−1^ to 1535 cm^−1^ post methanol crosslinking (Figure 5A) and additional peaks at 1630 cm^−1^ C=O stretch (amide I) and 1265 cm^−1^ C-N stretch, C-C stretch, and N-H bend (amide III) in comparison to SF scaffolds before methanol treatment, signifying the crystalline β sheet tertiary conformation. The CD-ECM post crosslinking to SF by methanol treatment as CD-ECM/SF hybrid scaffolds exhibited the SF β sheet conformation, at 1655, 1550, 1250, and 3330 cm^−1^ for type II collagen and at 1545 cm^−1^ for proteoglycans. The C-H stretch at 2847 cm^−1^ and 2915 cm^−1^ indicated the alkane group formation by alkene groups due to crosslinking of CD-ECM to the SF scaffolds (Figure 5B).

### 2.4. Biochemical Analysis and Morphological Characterization of Calcium Phosphate Crystallization by SEM/EDX

**Figure 6:**
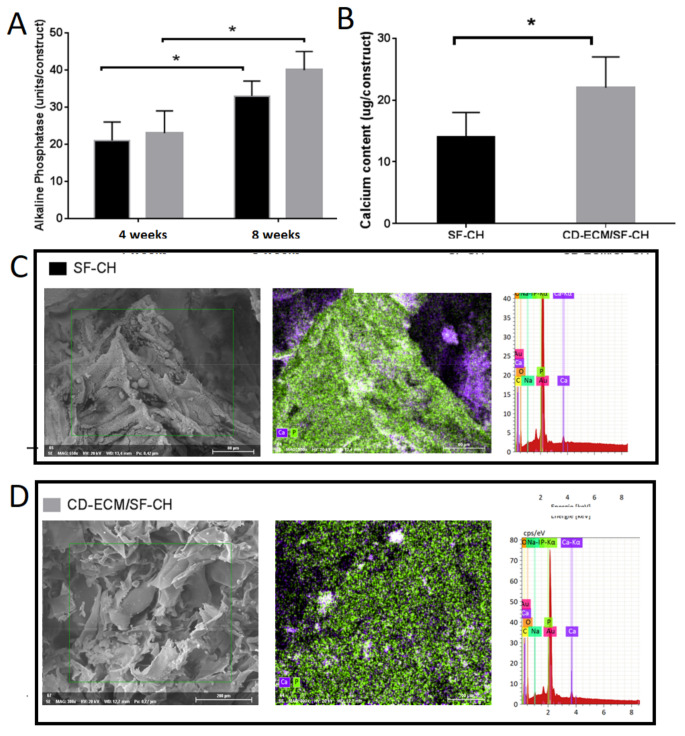
Alkaline phosphatase activity (**A**) of hypertrophic chondrocytes and calcium content (**B**) in the constructs at eight weeks post hypertrophic induction and calcium phosphate crystal, denoting cell-mediated mineralization in SF-CH (**C**) and CD-ECM/SF-CH (**D**) observed by scanning electron microscopy (SEM) and energy dispersive x-ray (EDX) analysis. Significant difference at * *p* < 0.05; *n* = 6. Scale bar represents 200 µm.

Biochemical analysis determined an increase in alkaline phosphatase levels secreted in both SF-CH and CD-ECM/SF-CH groups from four to eight weeks significantly (*p* < 0.05), but no differences were found between the groups. The calcium content in CD-ECM/SF-CH was significantly higher than in the SF group. Electron scanning electron microscopy (SEM) images showed, within the extracellular matrix, sphere-like structures identified as calcium phosphate crystal nodules morphologically, which was further confirmed by energy dispersive X-ray analysis (EDX) (Figure 6).

### 2.5. Hypertrophy and Osteogenic Gene Expression Post Differentiation towards Endochondral Ossification

**Figure 7:**
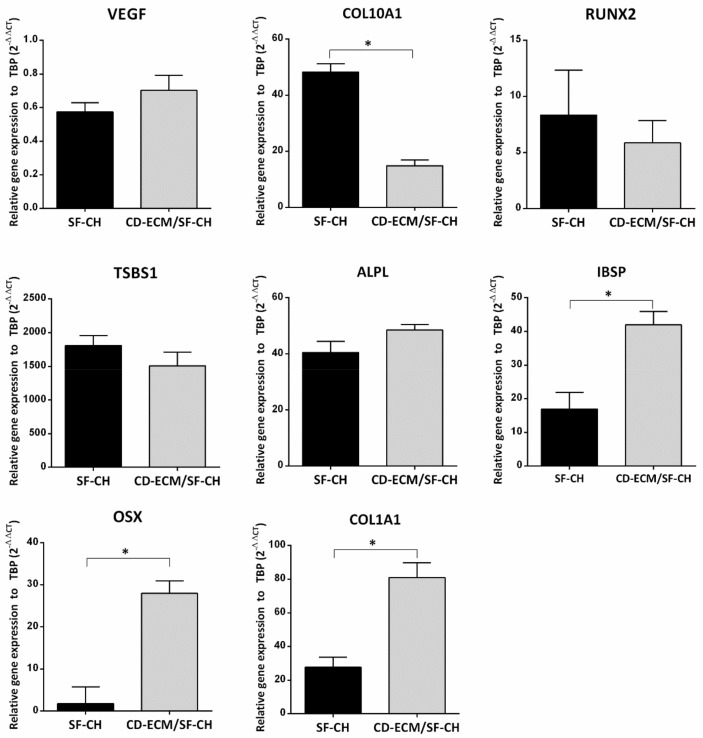
Gene expression for hypertrophic (VEGF, COL10A1, RUNX2) and osteogenic (ALPL, IBSP, OSX, COL1A1) markers in SF-CH and CD-ECM/SF-CH groups reveal a late hypertrophy stage indicated by upregulation of Thrombospondin (TSBS1). Significant difference at * *p* < 0.05; *n* = 6.

Quantitative RT-qPCR indicated that hypertrophic marker COL10A1 was significantly increased three-folds higher in the SF-CH group than in the CD-ECM/SF-CH group (*p* < 0.05). No significant differences were observed in VEGF, RUNX2, Tor SBS1. Whereas osteogenic markers IBSP was two-folds higher, OSX was 15-folds higher, and COL1A1 was three-folds higher in the CD-ECM/SF-CH group than in the SF-CH group with a significant difference (*p* < 0.05). No significant differences were observed for ALPL between the groups (Figure 7).

### 2.6. µCT Scanning and Biomechanical Tests

**Figure 8:**
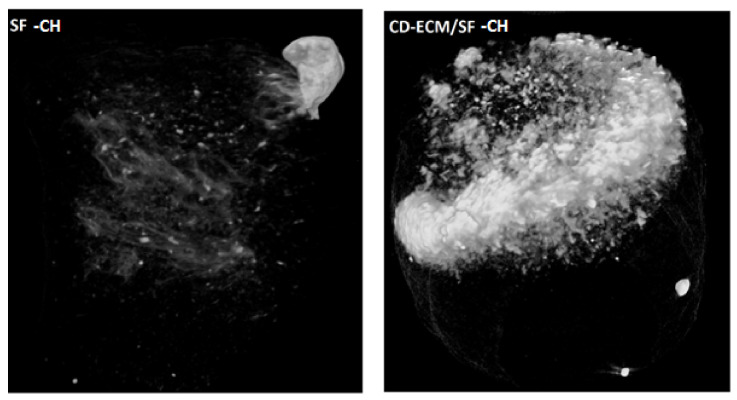
µCT scans of SF-CH (left) and CDECM/SF-CH (right) scaffolds after eight weeks of static culture.

The bone volume/total volume (BV/TV) calculated based on micro-CT scans for the SF-CH group was 0.92% and was twice the amount of 1.86% in CD-ECM/SF-CH group (Figure 8).

**Figure 9:**
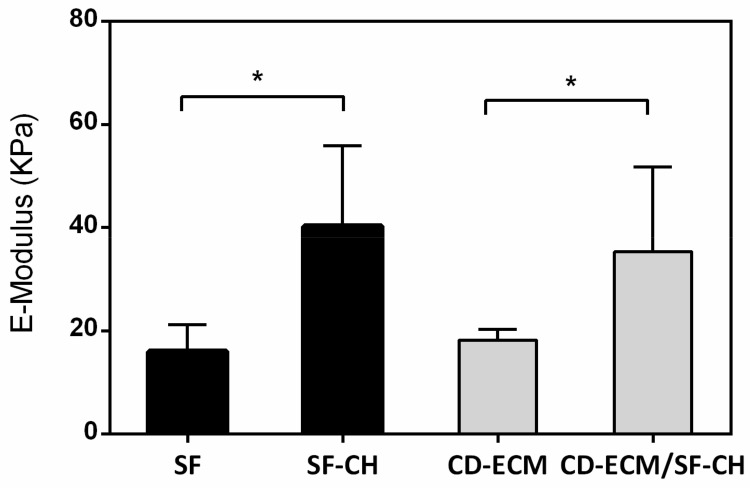
Biomechanical test results of empty SF, CD-ECM scaffolds compared to SF-CH, CD-ECM/-CH scaffolds with cells after eight weeks of static culture. Significant difference at * *p* < 0.05; *n* = 6.

Biomechanical tests revealed that the maximum force resulted from compressing the scaffold was up to 3 N. The compressive modulus of the scaffolds reached a maximum of 60 KPa for scaffolds seeded with BMSC’s differentiated to hypertrophic chondrocyte. SF scaffold showed similar mechanical properties to the hybrid scaffolds of CD-ECM (Figure 9). The compressive elastic modulus of both scaffold types loaded with MSC’s significantly increased from 22.01 ± 12.74 kPa for the control scaffold to 41.90 ± 14.87 kPa for SF scaffolds and from 18.02 ± 1.48 kPa to 40.24 ± 9.94 for CD-ECM/SF scaffolds after eight weeks of culture. The compressive elastic moduli of both SF and CD-ECM/SF scaffolds increased significantly in vitro. The differentiation and proliferation of hBMSC’s strengthened the compressive elastic moduli of scaffolds.

## 3. Discussion

The present study contemplated to develop an in vitro model of bone regeneration mimicking the endochondral ossification process by modulating the extracellular environment in tissue-engineered scaffolds. We investigated this approach by application of a decellularized cartilage-derived extracellular matrix (CD-ECM) onto silk fibroin (SF) as hybrid scaffolds (CD-ECM/SF), with appropriate mechanical properties to favor mineralization of human BMSCs. We found that incorporation of CD-ECM to SF scaffolds had a significant effect on early and late hypertrophy state of differentiation of hBMSC’s and expression of osteogenic markers compared to the same groups cultured without application of CD-ECM. The results indicate that the osteogenic potential, based on calcium phosphate (CaP) crystal nucleation and mineralized tissue formation of hBMSCs, was constructively increased by utilizing chondrogenic priming, followed by hypertrophic priming, and it was sufficient with no further osteogenic induction being required.

The FTIR findings exposed the effectiveness of methanol treatment for the deposition of CD-ECM to SF scaffold’s void struts by alkene to alkane transitional cross-linking process as retention of the ECM in a scaffold over culture period, and is vital for cell proliferation and for providing signals for cell differentiation. In this study, other than providing an external ECM environment, chondrogenic and hypertrophic factors were supplemented as biochemical cues to direct hBMSC’s towards the specific lineage, but in the case of an in vivo setting, such factors are not present, and the scaffold would stand alone moderating the cells from external cues [13,14]. Porosity in the scaffolds was controlled by a particle leaching method between 200 µm to 300 µm, as it was shown that vascularization and osseointegration occur only in bone grafts with pore size more than 200 µm upon implantation for critical size bone defects [15].

Morphological observation post eight weeks of differentiation by ESEM revealed that the chondrocytes were enlarged in size, indicating a differentiated state of hypertrophic chondrocytes. EDX measurements performed in the same area denoted a higher accumulation of CaP in the CD-ECM/SF-CH group than in the SF-CH group. This was further confirmed by biochemical analysis that calcium content was higher in the CD-ECM/SF-CH, followed by an increase in alkaline phosphatase levels secreted by the cells. Fetal calf serum supplementation in culture media was reported to contribute towards CaP deposition in silk fibroin scaffolds [16,17]; however, in our study, serum-free media were utilized to reduce osteogenic growth factors, permitting us to examine the in vitro mineralization ability of hBMSC differentiated hypertrophic chondrocytes.

Hypertrophic chondrocyte mediated mineralization assessed by RT-qPCR for hypertrophic and osteogenic markers revealed that COL10A1 gene expression was higher in the SF-CH group than the CD-ECM/SF-CH group, as in the CD-ECM/SF-CH group the decrease in COL10A1 could have occurred due to remodeling of the cartilaginous template by metalloproteinases towards mineralized tissue formation [18,19]. However, only in the CD-ECM/SF-CH group, it decelerated the cartilage template’s maturation and remodeling of the matrix, confirmed by an increased expression of early osteogenic markers ALP, IBSP, OSX, COLIA1, than in the SF-CH group indicating a precursor osteoblast state of differentiation. The late hypertrophic marker TSBS1 was expressed. The early hypertrophic marker CHM1 was absent; this differentiation towards a late hypertrophy stage along VEGF being defined would expedite vascular invasion onto the grafts followed by osseointegration upon implantation in vivo.

Micro-computed tomography (µCT) offers a non-destructive approach to the visualization of hydroxyapatite formation in tissue-engineered scaffolds [20] than conventional histological and calcium assays in bone tissue engineering; mineralized tissue formation is deemed to the final product of an in vitro cell culture. µCT scans demonstrated a dense cloud of hydroxyapatite minerals occurring on the CD-ECM/SF-CH group’s surface, whereas in the SF-CH group, it was comparatively less observed. However, the uniform distribution of hydroxyapatite was not observed throughout the scaffold. This could be attributed to the fact that static culture is inefficient in propagating the mineralized ossicles during ECO. On the contrary, dynamic culture systems such as flow perfusion enhanced hypertrophic matrix production but inhibited further bone formation [20,21]. Recent studies investigated mimicking the right biomechanical cues favoring ECO, such as hydrostatic pressure and chondrogenic priming enhanced hypertrophic matrix productions and mineralized matrix formation [22]. Similarly, the higher magnitude of cyclic tensile strain-induced ECO applies to chondrogenically primed MSC’s in fibrin hydrogels [23].

## 4. Materials and Methods

### 4.1. Decellularization and Isolation of Cartilage Derived Extracellular Matrix (CD-ECM)

Bovine knee joints were obtained from the local abattoir proceeding sacrifice of animals. The articular cartilage from both medial and lateral sides of the knee joint was collected with scalpels. The cartilage pieces were minced into fine pieces and frozen at −20 °C and lyophilized overnight. The freeze-dried cartilage was later coarsely ground in a cryomill (Retsch, Germany) for two cycles. The cryogrind powder was decellularized by osmotic shock, detergent, and enzymatic washing under constant agitation at 200 rpm. The ground cartilage powder was subjected to a hypertonic salt solution (HSS) for 12 h to disrupt the cell membrane. After HSS treatment, the cartilage was subjected to two cycles of Triton-X 100 (0.05% *v*/*v*) and HSS treatments to break down cellular membranes. The cartilage was further treated with benzonase (0.0625 KU/mL) for 12 h at 37 °C to fragment the nucleic acids. Sodium-lauroyl sarcosine (NLS, 1% *v*/*v*) was added for the next 12 h to remove the cells. Then, the cartilage was rinsed with 40% ethanol, and anionic exchange chromatography was performed to remove the organic solvents. The tissue was further rinsed with deionized water before freezing. Post decellularization, the tissue was lyophilized for 48 h and cryo-ground into a fine powder with a cryo-mill.

### 4.2. Fabrication of Cartilage Derived Extracellular Matrix and Silk Fibroin Hybrid (CD-ECM/SF) Scaffolds

Lyophilized SF was produced as previously described [24]. Briefly, cocoons of *B. mori* (Seidentraum, Germany) were boiled in 0.02 M Na_2_CO_3_ twice for 1 h, and the resulting fibers were rinsed five times in ddH_2_O and dried overnight. The fibers were dissolved in 9.3 M LiBr and dialyzed for three days against ddH_2_O. The resulting regenerated silk fibroin solution was lyophilized until further usage. Lyophilized CD-ECM and SF were dissolved in 1,1,1,3,3,3-Hexa- fluoro-2-propanol (HFIP, Sigma-Aldrich, St. Louis, MO, USA) at a ratio of 1:1 to yield a 17% (w/v) solution and cast onto a teflon mold. The resulting scaffolds were cut with a dermal biopsy punch in a dimension of 5 mm h, 5 mm d. The cut scaffolds were immersed in 90% methanol to induce confirmation of secondary structure of β sheets in SF. Post-washes in PBS and sterilization were achieved by autoclaving at 121 °C, and the scaffolds were used for cell culture.

### 4.3. Cell Culture and Hypertrophic Differentiation of BMSC’s

Human bone marrow derived MSC’s (hBMSCs,) were purchased from Lonza (Lonza, Switzerland). After expansion to passage twp, cells were seeded onto the scaffold’s surface at an initial seeding density of 1 × 10^6^ cells in 20 µL suspensions and let incubated at 37 °C for 2 h. Post seeding, the scaffolds were immersed for the next 24 h in 10% FCS containing basal medium (DMEM F12, GLUTAMAX, 1% ITS, 1.5 mg/mL BSA, 2% penstrep, 2.5 µg/mL amphob) for the cells to be at an equilibrium. The constructs are primed towards chondrogenesis for the next two weeks in serum-free basal medium (1% ITS, 1% BSA, 25 mM HEPES, DMEM high glucose) with chondrogenic supplements (100 nM Dexamethasone, 50 µg/mL Ascorbic-acid-2-phosphate, 10 ng/mL TGF β1). After two weeks, the constructs are primed towards hypertrophic chondrocytes with serum-free basal media and hypertrophy supplements (1nM Dexamethasone, 50 µg/mL Ascorbic-acid-2-phosphate, 50 ng/mL L-Thyroxine, 10mM β-Glycerolphosphate) for the next six weeks under static conditions.

### 4.4. Biochemical Analysis

#### 4.4.1. sGAG and DNA Assays

Total sGAG was measured from decellularized cartilage (CD-ECM) by freezing them at −80 °C and lyophilized at 20 °C to evaporate the water content. Quantification was done by treating them overnight with 25u/mL proteinase K enzyme at 56 °C. Enzyme inactivation was then performed at 90 °C for 10 min. The resultant supernatant was transferred to ultra-free filter reaction tubes of 0.1 µm pore size (Millipore, Billerica, MA, USA) and centrifuged at 12,000× *g* for 10 min. sGAG was measured through complexation and decomplexation with a 1,9 dimethyl methylene blue solution (DMMB). The absorbance was measured at 656 nm in a plate reader. For DNA analysis, *n* = 6 constructs after the decellularized cartilage (CD-ECM) were compared to the native articular cartilage. Briefly, the supernatant above from the Proteinase K digested constructs were removed before ultrafiltration. The DNA content was measured fluorometrically using the PicoGreen assay (Fisher Scientific), according to the manufacturer’s instructions at an excitation wavelength of 480 nm, emission wavelength of 528 nm.

#### 4.4.2. Hydroxyproline Assay

Total collagen content was determined by quantifying the hydroxyproline content. CD-ECM and native cartilage samples were hydrolyzed in 6 M HCL at 110 °C for 18 h, and the hydroxyproline content from the hydrolyzed solution was measured with a chloramine-T/Ehrlich spectrophotometry-based assay (Sigma-Aldrich, St. Louis, MO, USA). The hydroxyproline content was measured at a wavelength of 560 nm.

#### 4.4.3. Alkaline Phosphatase Assay

Extracellular alkaline phosphatase (ALP) production was determined from the supernatant of cells based on a custom-made biochemical assay in all the cell-scaffold constructs. The samples were briefly measured colorimetrically by converting p-nitrophenyl phosphate to p-nitrophenol in a spectrophotometer at 405 nm.

#### 4.4.4. Calcium Assay

Calcium mineralization was quantitatively determined in cell-scaffold constructs by disintegrating them in a mechanical homogenizer (MagnaLyser) in 5% trichloroacetic acid. The resulting supernatant was measured spectrophotometrically at 575 nm based on the reaction with o-cresolphthalein complexion, according to manufacturer’s instructions (Sigma-Aldrich, St. Louis, MO, USA).

### 4.5. Scanning Electron Microscopy (SEM) and Energy Dispersive X-ray (EDX) Analysis

Cell attachment and distribution after eight weeks were observed using a Hitachi ESEM-FEG. Cell-scaffold constructs were rinsed twice with PBS and fixed in 10% formalin for 2 hr. Subsequently, the samples were cut in progressive sections and dehydrated in sequential ethanol series (50%, 60%, 70%, 80%, 90%, 96%, and 100%), 30 min for each concentration. For the final dehydration step, scaffolds were immersed in hexamethyldisilazane, and the solvent was left to evaporate overnight. Finally, samples were gold sputter costed prior to SEM analysis. SEM images were obtained under a high vacuum with an acceleration voltage of 10 kV and a working distance of 25 mm. An EDX (Bruker) system integrated into the SEM was expended to analyze the possible co-localization of calcium (Ca) and phosphate (P) in the extracellular matrix. The spectra were taken under a high vacuum with an acceleration voltage of 10 kV and a working distance of 10 mm.

### 4.6. Attenuated Total Reflection Fourier-Transform Infrared Spectroscopy (ATR-FTIR)

ATR-FTIR spectra were collected using a Spectrum Two FT-IR Spectrometer (PerkinElmer Inc.) equipped with a LiTaO3 detector and with a MIRacl single reflection ATR (ZnSe) accessory (PIKE Technologies). Additionally, 4 to 64 scans were co-added at a nominal resolution of 4 cm^−1^. After each data acquisition, ATR correction was performed; for all spectral manipulation, the Spectrum 10 software package (PerkinElmer Inc.) was used.

### 4.7. Real-Time Quantitative PCR

Cell-scaffold constructs were minced into fine pieces in lysis buffer and added to the MagNA Lyser tubes. The tubes were frozen in liquid nitrogen and homogenized with the MagNA Lyser four times at 6500 rpm. The samples were treated by Proteinase K for 30 min at 55 °C and centrifuged to pellet the debris. The resulting supernatant was used for RNA isolation with the Qiagen Eneasy Fibrous Tissue Kit, followed by elution with 30 µl of RNase-free water. cDNA synthesis was performed using Transcriptor First Strand cDNA Synthesis Kit (Roche, Basel, Switzerland), and additionally, RNA from bacteriophage MS2 was added to stabilize the isolated RNA during cDNA synthesis. Probe-based real-time quantitative polymerase chain reaction (RTqPCR) was performed in triplicates in the LightCycler 96 using Fast Start Essential DNA Probes Master. (Roche, Basel, Switzerland). Probe-primer pairs were designed for hypertrophic and osteogenic specific markers COLX, RUNX2, TSBS1, CHM1, VEGF, COLI, ALPL, IBSP, OSX (Table 1),with IDT Real-Time qPCR software and synthesized by IDT (Integrated DNA Technologies, Leuven, Belgium). As a reference gene, we chose TBP [25] Annealing temperature, which was experimentally determined for reference. Each target gene and the relative expression was evaluated with the 2^−∆∆Ct^ method.

### 4.8. Biomechanical Analysis

Cell-scaffold constructs were tested under compression load using the Zwick test machine (ZwickRoell GmbH, Ulm, Germany), equipped with a 10 N load cell and carried out with a 1 mm/min cross-head speed. All samples were wet during testing. The displacement was tracked using a three-dimensional Digital Image Correlation (DIC) system (GOM GmbH, Germany) equipped with two CCD cameras (6 Megapixels). Thirty scaffolds with a 5 mm diameter and 5 mm in height were evaluated. The compressive force-displacement curves were converted into stress and strain curves. Compressive elastic moduli were calculated from the slopes of the stress−strain curves. The average compressive modulus and standard deviation of different sample groups were calculated.

### 4.9. Micro-Computed Tomography (µCT) Analysis

The wet constructs were scanned with a calibrated micro CT scanner (Bruker Skyscan 1173, Bruker, Kontich, Belgium) at 30 kV, 180 μA, integration time 3200 ms, nominal resolution of 5 μm, and without a filter. Scaffolds were scanned in wet conditions, except empty scaffolds for porosity analysis were scanned dry. Using medtool (Version 4.3; Dr. Pahr Ingenieurs e.U., Pfaffstätten, Austria), the µCT scans were processed. First, raw image files from Brucker were imported into Medtool, and the region of interest was cropped. Second, registration was performed using the iterative selection method to find a single level threshold at the minimum between the scaffold constructs and calcified regions. Third, the equivalent bone volume to total volume was calculated as the number of calcified voxels divided by the voxels’ total number in one scaffold.

### 4.10. Statistical Analysis

A non-parametric Mann–Whitney two-tailed U-test was performed for comparisons between two datasets at a time. Multiple comparisons were performed using the non-parametric Kruskal–Wallis test followed by Dunn’s multiple comparisons test. All data are presented as the mean ± SEM. The significance level was set at *p* < 0.05. All statistical analyses were performed using the GraphPad Prism software (Graphpad Prism Software Inc., San Diego, CA, USA).

## 5. Conclusions

The results from this study revealed that incorporation of a decellularized cartilaginous template onto silk fibroin scaffolds has a significant effect on hypertrophy-mediated osteogenic differentiation of hBMSC’s. Precisely, this study’s results also show that there was a reduction in early hypertrophy markers and an increase in late hypertrophic markers, a foremost constraint to existing bone tissue engineering approaches only by applying osteogenic induction alone. Likewise, the present study’s results showed an acceleration of hBMSC-mediated mineralized tissue formation in the hybrid scaffolds, post hypertrophic induction. We developed an in vitro model of endochondral ossification intermediated developmental tissue engineering approach for bone regeneration.

## Figures and Tables

**Table 1:** List of human primers used in this study.

Name	Identification	Forward	Reverse	Annealing t°
TBP	NM_003194	GAGAGTTCTGGGATTGTACCG	ATCCTCATGATTACCGCAGC	58
COL1A1	NM_000088	CCCCTGGAAAGAATGGAGATG	TCCAAACCACTGAAACCTCTG	62
Col10A1	NM_000493	CAAGGCACCATCTCCAGG	TGGGCATTTGGTATCGTTCAG	58
ALPL	NM_000478	GATGTGGAGTATGAGAGTGACG	GGTCAAGGGTCAGGAGTTC	59
CHM1	NM_007015	TGGAAATAGACGCTGGGAAC	GCCTTCACTTGCGCTTTAATG	60
RUNX2	NM_001015051	TTCACCTTGACCATAACCGTC	GGCGGTCAGAGAACAAACTAG	61
IBSP	NM_004967	ATTTCCAGTTCAGGGCAGTAG	GTGTGGTATTCTCAGCCTCAG	58
TSBS1	NM_003246	TCTCTGACCTGAAATACGAATGTAG	AAGGAAGCCAAGGAGAAGTG	62
OSX	NM_152860	CCAGCAACCCCAGAGAAAG	TTGGCAAGCAGTGGTCTAG	62
VEGF A	NM_003376	AGTCCAACATCACCATGCAG	TTCCCTTTCCTCGAACTGATTT	63

## Data Availability

The datasets corresponding to the current study are available from the corresponding author upon request.
